# Artificial intelligence–based prognostic modeling of immunoradiotherapy in Barcelona clinic liver cancer stage C hepatocellular carcinoma: a multicenter retrospective study

**DOI:** 10.3389/fonc.2026.1784711

**Published:** 2026-03-05

**Authors:** Ying-Jie Li, Lei Yang, Su Li, Shuo Chen, Yuan-Ping Zhong, Lianbin Wen, Yanqiong Song, Yuan Li

**Affiliations:** 1 Department of General Surgery, Hospital of Sichuan College of Traditional Chinese Medicine, Mianyang, Sichuan, China; 2 Department of General Surgery, Mianyang Hospital of Traditional Chinese Medicine, Mianyang, Sichuan, China; 3 Department of Anesthesiology, Hospital of Sichuan College of Traditional Chinese Medicine, Mianyang, Sichuan, China; 4 Department of Anesthesiology, Mianyang Hospital of Traditional Chinese Medicine, Mianyang, Sichuan, China; 5 Department of Critical Care Medicine, Mianyang Central Hospital, University of Electronic Science and Technology of China, Mianyang, Sichuan, China; 6 Department of Anesthesiology, Affiliated Hospital of Zunyi Medical University, Zunyi, Guizhou, China; 7 Department of Geriatric Cardiology, Sichuan Academy of Medical Sciences and Sichuan Provincial People’s Hospital, Chengdu, China; 8 Department of Radiotherapy, Sichuan Cancer Hospital and Institute, Sichuan Cancer Center, School of Medicine, University of Electronic Science and Technology of China, Chengdu, China

**Keywords:** artificial intelligence, BCLC, hepatocellular carcinoma, immunoradiotherapy, prognostic modeling

## Abstract

**Background:**

Barcelona Clinic Liver Cancer (BCLC) stage C hepatocellular carcinoma is associated with poor prognosis, and conventional systemic therapies offer limited survival benefit. Immunotherapy combined with radiotherapy has emerged as a promising approach, but patient responses are heterogeneous. Artificial intelligence (AI) may facilitate individualized prognostic prediction to guide therapy.

**Methods:**

We retrospectively analyzed 198 BCLC stage C HCC patients from three centers. The experimental group received immunoradiotherapy plus targeted therapy, and the control group received immunotherapy plus targeted therapy. Baseline characteristics were balanced using inverse probability of treatment weighting (IPTW). Five machine learning models (Cox, LASSO, DT, RSF, and XGBoost) were developed to predict 6-, 12-, and 24-month overall survival.

**Results:**

Before and after IPTW adjustment, the experimental group showed longer progression-free and overall survival than the control group. In the training cohort, the RSF model achieved the highest concordance index (0.7458). In the validation cohort, it also demonstrated the best receiver operating characteristic – area under the curve (ROC-AUC) values for 6-, 12-, and 24-month OS (0.821, 0.818, and 0.791, respectively). Decision curve analysis and calibration plots indicated good stability. Variable importance analysis showed that tumor number, tumor size, and portal vein tumor thrombosis consistently contributed substantially to survival prediction across all time points.

**Conclusions:**

Immunoradiotherapy represents a promising therapeutic option for BCLC stage C HCC. The RSF-based model may support individualized prognostic risk stratification and clinical decision-making.

## Background

Hepatocellular carcinoma (HCC) is the predominant primary liver malignancy and a leading cause of cancer-related mortality worldwide (1). Globally, more than 900000 new cases are diagnosed annually, with the highest incidence observed in Asia and sub-Saharan Africa (2, 3). Chronic liver diseases, including hepatitis B or C virus infection, alcohol-related liver injury, and non-alcoholic fatty liver disease, are major risk factors (4, 5). Despite improvements in early detection and localized interventions, most patients present with advanced-stage disease at diagnosis, limiting therapeutic options and contributing to poor overall survival (6).

Patients classified as Barcelona Clinic Liver Cancer (BCLC) stage C HCC typically exhibit vascular invasion, extrahepatic metastases, or impaired liver function, resulting in a dismal prognosis (7–9). Conventional systemic therapies and targeted treatments provide only modest survival benefits (10, 11). In recent years, immunotherapy, particularly PD-1/PD-L1 inhibitors, combined with radiotherapy has emerged as a promising strategy for advanced HCC (12–15). This combination may enhance tumor antigen presentation, stimulate cytotoxic T-cell responses, and even induce abscopal effects at distant non-irradiated sites. Nevertheless, clinical responses are highly heterogeneous, and reliable predictive markers for individual outcomes remain elusive (16–18).

Artificial intelligence (AI) offers a powerful approach to address these challenges by enabling the analysis of complex, multidimensional biomedical data. Machine learning (ML) models, in particular, can integrate imaging features, clinical parameters, and molecular data to generate individualized prognostic predictions (19–21). Compared with traditional statistical methods, ML algorithms are better equipped to capture nonlinear and high-dimensional relationships, improving predictive performance in survival analysis and treatment response assessment across various malignancies (22, 23). In the context of advanced HCC, AI-driven models could help identify high-risk patients, guide therapeutic decision-making, and support personalized immunoradiotherapy strategies (24).

In summary, BCLC stage C HCC remains associated with extremely poor survival outcomes, while immunoradiotherapy represents a potentially effective yet variable treatment option. The application of AI, especially machine learning, provides a novel framework to predict prognosis and optimize therapy for these patients. Investigating AI-based prognostic models in this setting may facilitate individualized treatment planning and contribute to the advancement of precision medicine in advanced HCC.

## Methods

We retrospectively collected data from 198 patients with BCLC stage C HCC across three Chinese hospitals. Patients were divided into two groups: the experimental group received combined immunoradiotherapy plus targeted therapy, while the control group received immunotherapy plus targeted therapy without radiotherapy. Patients with missing baseline variables or incomplete follow-up/outcome information were excluded prior to analysis.

Inclusion criteria were: (1) diagnosis of BCLC stage C HCC, (2) Child–Pugh class A or B liver function, and (3) availability of follow-up data. Exclusion criteria included: (1) Child–Pugh class C, (2) incomplete clinical or treatment data, and (3) inability to receive radiotherapy.

Given that the data used were fully anonymized with no possibility of identifying individual patients and the study did not interfere with any clinical treatment decisions of the patients, ethical approval was waived by the Mianyang Hospital of Traditional Chinese Medicine. All patients had signed written informed consent forms prior to receiving clinical treatment, in accordance with the principles of the Declaration of Helsinki.

### Follow-up and outcomes

The primary endpoint of this study was overall survival (OS), defined as the time from initiation of treatment to death from any cause or last follow-up. The secondary endpoint was progression-free survival (PFS), defined as the time from treatment initiation to the first documented disease progression or death, whichever occurred first. Disease progression was assessed according to modified RECIST (mRECIST) for HCC based on routine follow-up contrast-enhanced CT and/or MRI. PFS events and dates were adjudicated by investigators at each center through review of medical records (including imaging reports) and were supplemented by telephone follow-up when necessary. Patients without progression were censored at the date of the last available clinical/imaging assessment or the last successful telephone contact.

### Radiotherapy benefit analysis and AI model construction

To evaluate the potential survival benefit of radiotherapy, inverse probability of treatment weighting (IPTW) was applied to balance baseline characteristics between the experimental and control groups. The PS model included baseline covariates: age, sex, tumor number, tumor size, HBV infection, Child–Pugh class, portal vein tumor thrombosis (PVTT), N stage, M stage, and alpha-fetoprotein (AFP). OS and PFS were compared before and after IPTW adjustment to assess the effect of radiotherapy.

For the construction of artificial intelligence-based prognostic models, univariate Cox regression was first used to screen candidate predictors associated with OS, using P < 0.05 as the selection threshold. Subsequently, patients were randomly divided into a training cohort (70%) and a validation cohort (30%) using a 7:3 random allocation. Model training and tuning were performed using 5-fold cross-validation in the training cohort. No standardization was applied to continuous variables. In the training set, five machine learning algorithms—including multivariate Cox model, LASSO regression, decision tree (DT), random survival forest (RSF), and XGBoost—were developed. The concordance index (C-index) was calculated for each model to evaluate discriminative performance.

Model validation was conducted in the validation set using receiver operating characteristic (ROC) curves, decision curve analysis (DCA), and calibration plots to assess predictive accuracy and clinical utility. In addition, variable importance plots were generated in the training cohort to interpret the contribution of each feature to the machine learning models, providing insight into the key prognostic factors influencing survival outcomes.

### Statistical analysis

Continuous variables were compared using the t-test, and categorical variables using the chi-square test. Kaplan–Meier curves for OS and PFS were compared with the log-rank test. All analyses were performed in R, and a two-sided p-value <0.05 was considered statistically significant.

## Results

### Baseline characteristics

Before IPTW adjustment, the control and experimental groups were generally comparable in age, sex, tumor characteristics, HBV infection, and Child–Pugh class. Notable differences included a higher prevalence of PVTT (85.9% *vs*. 70.8%, p=0.017) and AFP ≥400 ng/mL (58.7% *vs*. 43.4%, p=0.045) in the control group (**Table 1**). After IPTW adjustment, baseline characteristics were well balanced between the control and experimental groups, with no significant differences observed (all p > 0.05, SMDs < 0.1, **Table 2**; **Supplementary Figure 1**).

**Table 1:** Baseline characteristics of patients in the control and experimental groups before IPTW.

Variable	Level	Control group (n=92)	Experimental group (n=106)	P value	SMD
Age (years)	Mean (SD)	53.91 (10.83)	53.75 (9.91)	0.910	0.016
Sex, n (%)	Female	16 (17.4)	14 (13.2)	0.535	0.116
	Male	76 (82.6)	92 (86.8)		
Tumor number, n (%)	1	28 (30.4)	37 (34.9)	0.606	0.095
	≥2	64 (69.6)	69 (65.1)		
Tumor size (cm), n (%)	<5	25 (27.2)	28 (26.4)	0.883	0.071
	≥5, <10	43 (46.7)	47 (44.3)		
	≥10	24 (26.1)	31 (29.2)		
HBV infection, n (%)	No	29 (31.5)	31 (29.2)	0.847	0.050
	Yes	63 (68.5)	75 (70.8)		
Child–Pugh class, n (%)	A	71 (77.2)	80 (75.5)	0.910	0.040
	B	21 (22.8)	26 (24.5)		
PVTT, n (%)	No	13 (14.1)	31 (29.2)	0.017	0.373
	Yes	79 (85.9)	75 (70.8)		
N stage, n (%)	No	44 (47.8)	50 (47.2)	1.000	0.013
	Yes	48 (52.2)	56 (52.8)		
M stage, n (%)	No	57 (62.0)	80 (75.5)	0.057	0.295
	Yes	35 (38.0)	26 (24.5)		
AFP (ng/mL), n (%)	<400	38 (41.3)	60 (56.6)	0.045	0.310
	≥400	54 (58.7)	46 (43.4)		

**Table 2:** Baseline characteristics of patients in the control and experimental groups after IPTW.

Variable	Level	Control group (n = 197.06)	Experimental group (n = 202.91)	P value	SMD
Age (years)	Mean (SD)	53.47 (10.74)	53.83 (9.91)	0.830	0.035
Sex, n (%)	Female	27.9 (14.2)	29.0 (14.3)	0.983	0.003
	Male	169.1 (85.8)	174.0 (85.7)		
Tumor number, n (%)	1	58.5 (29.7)	62.4 (30.8)	0.880	0.023
	≥2	138.5 (70.3)	140.5 (69.2)		
Tumor size (cm), n (%)	<5	62.6 (31.8)	58.0 (28.6)	0.909	0.069
	≥5, <10	83.5 (42.4)	90.5 (44.6)		
	≥10	51.0 (25.9)	54.4 (26.8)		
HBV infection, n (%)	No	54.7 (27.7)	61.9 (30.5)	0.700	0.061
	Yes	142.4 (72.3)	141.0 (69.5)		
Child–Pugh class, n (%)	A	154.9 (78.6)	158.7 (78.2)	0.950	0.009
	B	42.2 (21.4)	44.2 (21.8)		
PVTT, n (%)	No	47.9 (24.3)	44.7 (22.0)	0.754	0.054
	Yes	149.1 (75.7)	158.2 (78.0)		
N stage, n (%)	No	85.9 (43.6)	91.9 (45.3)	0.826	0.035
	Yes	111.2 (56.4)	111.0 (54.7)		
M stage, n (%)	No	137.9 (70.0)	135.8 (66.9)	0.681	0.066
	Yes	59.1 (30.0)	67.1 (33.1)		
AFP (ng/mL), n (%)	<400	98.5 (50.0)	96.4 (47.5)	0.758	0.049
	≥400	98.6 (50.0)	106.5 (52.5)		

### Survival benefit of radiotherapy

**Figure 1:**
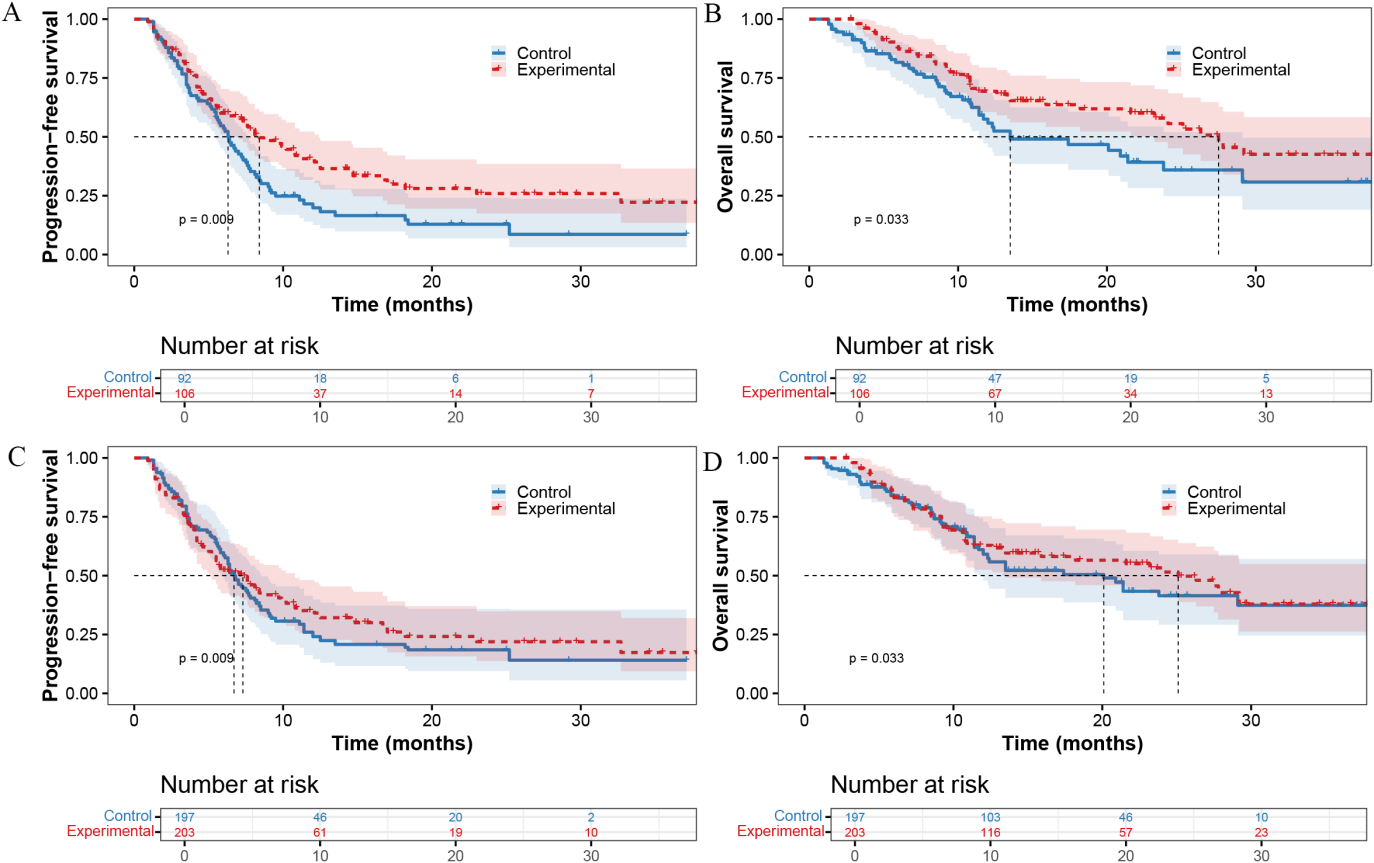
Survival outcomes before and after inverse probability of treatment weighting (IPTW). **(A)** Progression-free survival before IPTW. **(B)** Overall survival before IPTW. **(C)** Progression-free survival after IPTW adjustment. **(D)** Overall survival after IPTW adjustment.

Before IPTW adjustment, patients in the experimental group exhibited significantly longer median PFS (mPFS, 8.4 *vs*. 6.3 months; p=0.009, **Figure 1A**) and median OS (mOS, 27.5 *vs*. 13.5 months; p=0.033, **Figure 1B**) compared with the control group. After IPTW adjustment, the experimental group still showed improved survival, with longer mPFS (7.3 *vs*. 6.7 months; p=0.009, **Figure 1C**) and mOS (25.1 *vs*. 20.1 months; p=0.033, **Figure 1D**).

**Figure 2:**
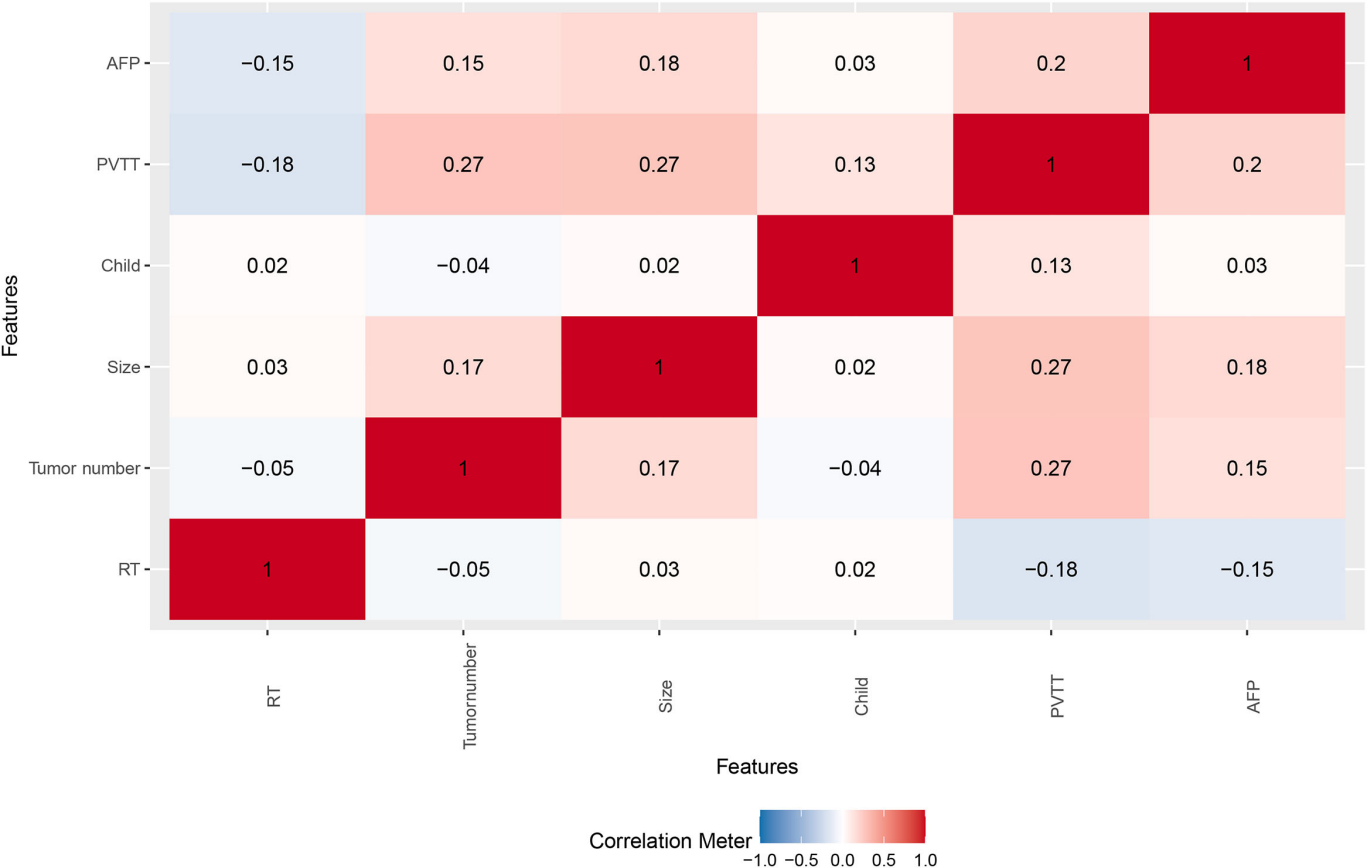
Pearson correlation heatmap of clinical features in the study cohort.

Univariable Cox analysis showed that radiotherapy was associated with improved OS (HR 0.63, 95% CI 0.41–0.97, p=0.035), while tumor number ≥2, tumor size ≥5 cm, Child–Pugh class B, PVTT, and AFP ≥400 ng/mL were associated with worse survival (**Table 3**). Correlation analysis indicated low inter-variable correlations among radiotherapy, tumor number, tumor size, Child–Pugh class, PVTT, and AFP, supporting their independent contribution to survival prediction (**Figure 2**).

**Table 3:** Univariable Cox proportional hazards regression analysis.

Variable	Category/Level	HR (95% CI)	P value
RT	No RT	Reference	
	RT	0.63 (0.41–0.97)	0.035
Age	Per year increase	1.01 (0.99–1.03)	0.512
Sex	Female	Reference	
	Male	1.29 (0.67–2.50)	0.449
Tumor number	1	Reference	
	≥2	1.92 (1.17–3.15)	0.009
Tumor size (cm)	<5	Reference	
	≥5, <10	2.36 (1.21–4.61)	0.012
	≥10	4.21 (2.14–8.31)	<0.001
HBV infection	No	Reference	
	Yes	1.59 (0.97–2.63)	0.068
Child–Pugh class	A	Reference	
	B	1.65 (1.01–2.70)	0.047
PVTT	No	Reference	
	Yes	3.17 (1.64–6.14)	<0.001
N stage	No	Reference	
	Yes	1.15 (0.75–1.76)	0.522
M stage	No	Reference	
	Yes	1.42 (0.92–2.18)	0.113
AFP (ng/mL)	<400	Reference	
	≥400	1.68 (1.09–2.59)	0.019

### Artificial intelligence model construction and validation

**Figure 3:**
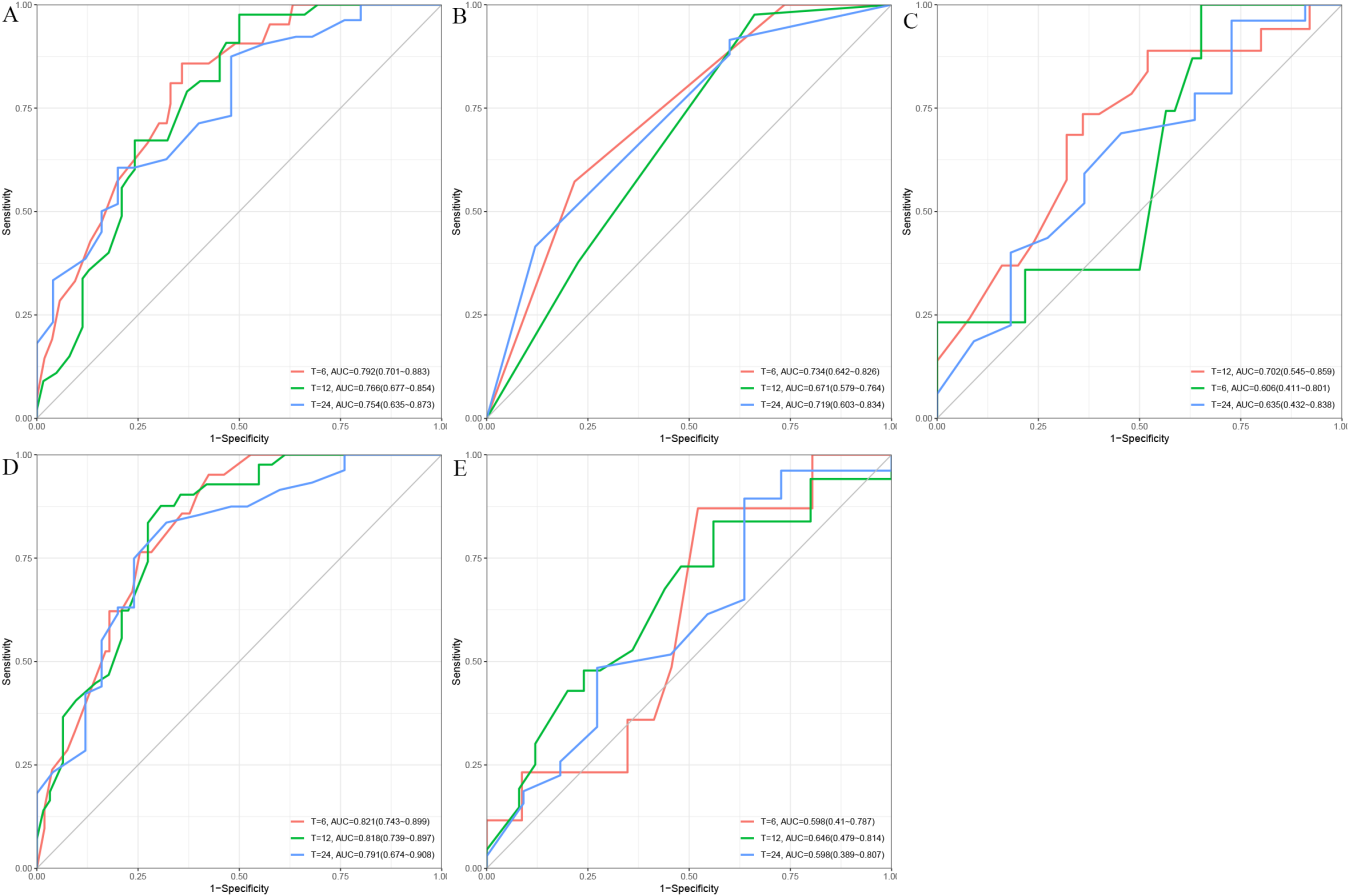
Time-dependent receiver operating characteristic (ROC) curves for five predictive models at 6, 12, and 24 months. **(A)** Cox proportional hazards model; **(B)** Lasso-penalized Cox regression; **(C)** Decision Tree (DT); **(D)** Random Survival Forest (RSF); **(E)** XGBoost.

Five machine learning algorithms—including multivariate Cox, LASSO regression, decision tree (DT), random survival forest (RSF), and XGBoost—were developed in the training cohort using radiotherapy, tumor number, tumor size, Child–Pugh class, PVTT, and AFP to predict 6-, 12-, and 24-month OS, with C-indices of 0.7248, 0.6607, 0.6319, 0.7458, and 0.6168, respectively. In the validation cohort, the receiver operating characteristic-area under the curve (ROC-AUC) values for 6-, 12-, and 24-month OS were 0.792, 0.766, and 0.754 for the Cox model (**Figure 3A**); 0.734, 0.671, and 0.719 for LASSO regression (**Figure 3B**); 0.702, 0.606, and 0.635 for DT (**Figure 3C**); 0.821, 0.818, and 0.791 for RSF (**Figure 3D**); and 0.598, 0.646, and 0.598 for XGBoost (**Figure 3E**).

**Figure 4:**
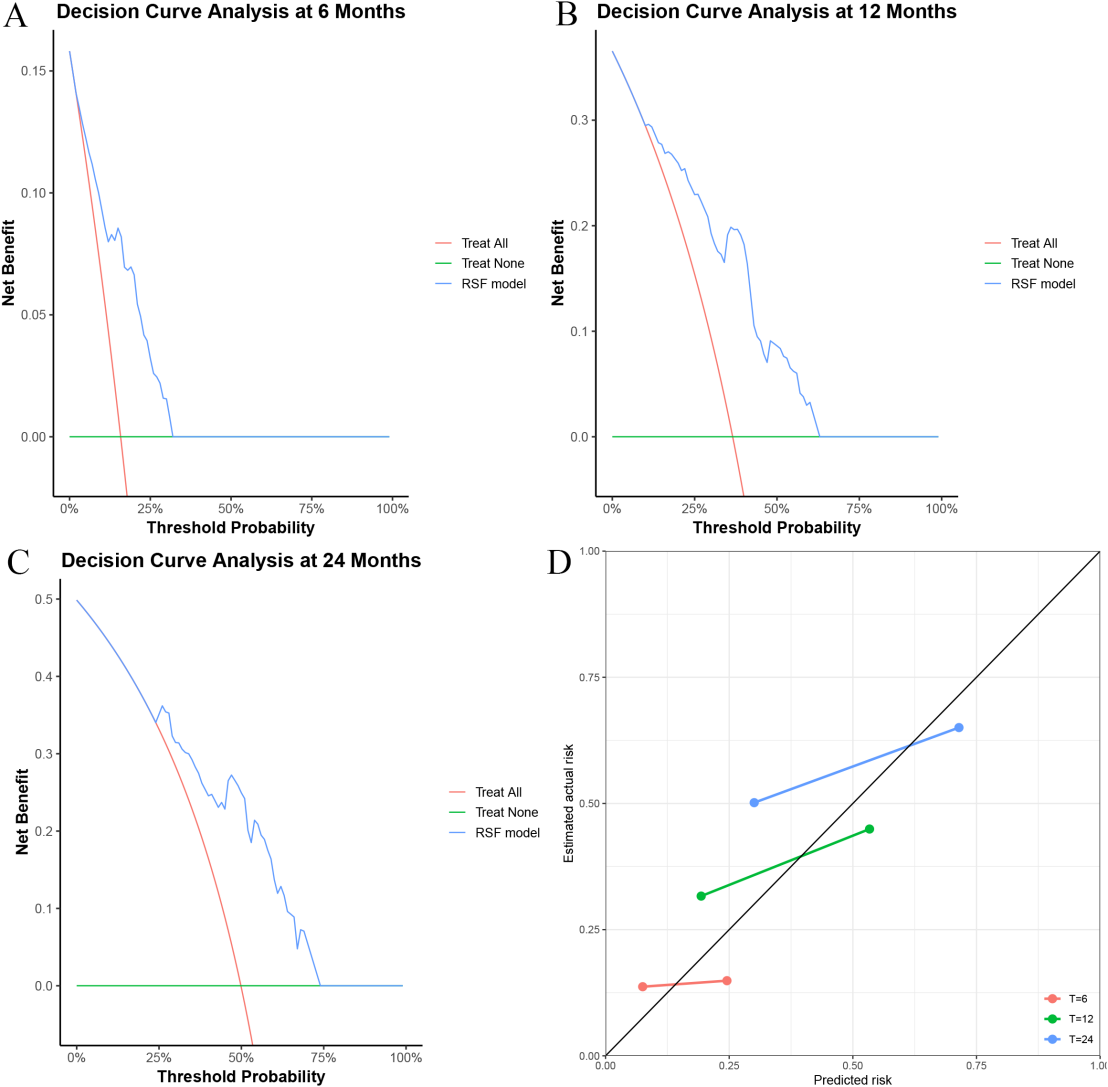
Clinical utility and calibration of the Random Survival Forest (RSF) model. Decision curve analysis at 6 **(A)**, 12 **(B)**, and 24 **(C)** months, respectively, showing the net benefit of the RSF model across a range of threshold probabilities. **(D)** Calibration plot comparing predicted probabilities with observed event rates at 6, 12, and 24 months.

DCA demonstrated stable predictive performance of the RSF model (**Figures 4A–C**). The calibration plots showed close overlap between the predicted and observed survival probabilities at 6, 12, and 24 months, indicating excellent calibration (**Figure 4D**).

**Figure 5:**
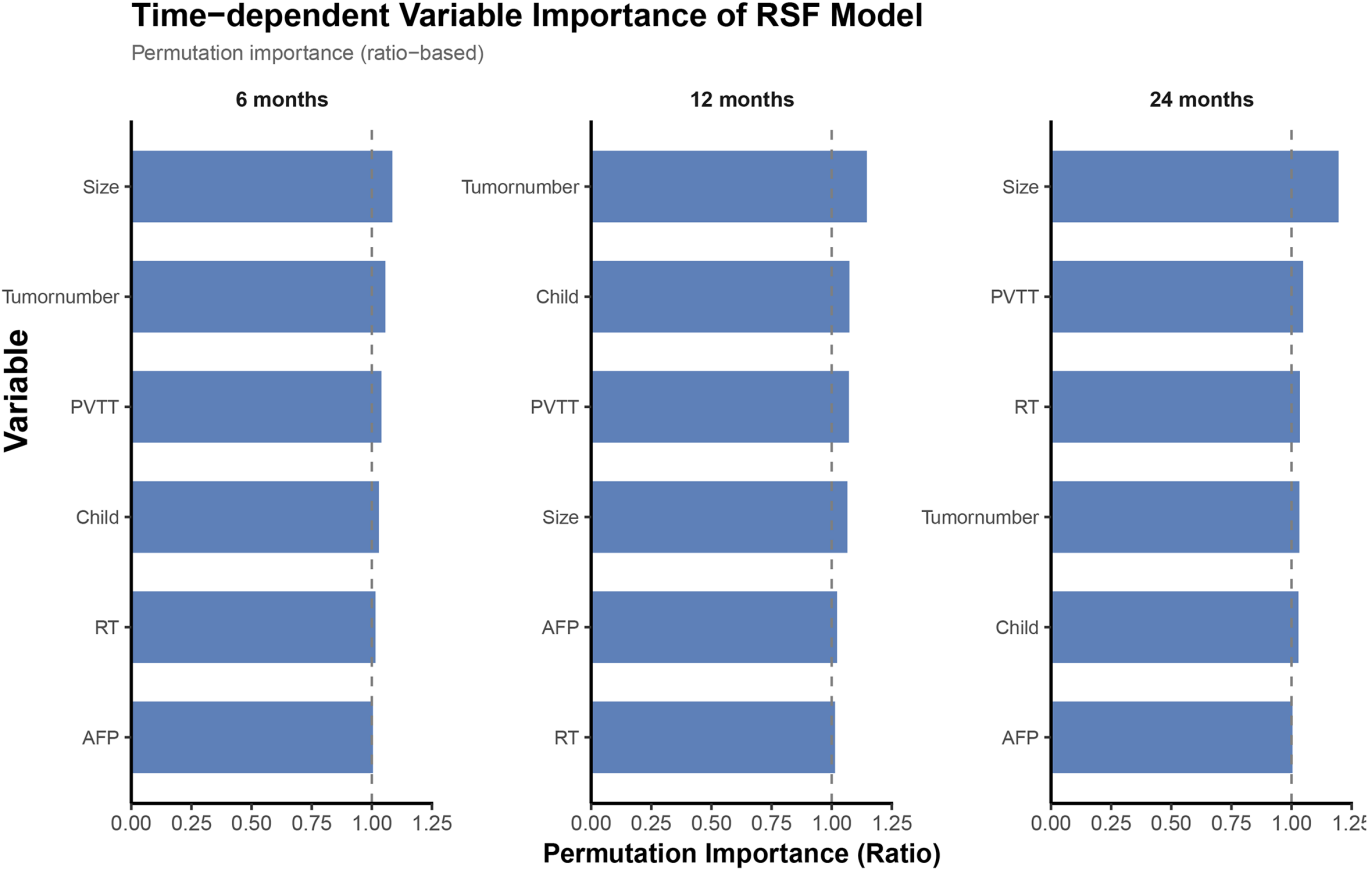
Variable importance from the Random Survival Forest (RSF) model at 6, 12, and 24 months, measured by permutation-based importance ratios. Higher values indicate greater contribution of a feature to predictive accuracy.

In the validation cohort, variable importance analysis showed that tumor number, tumor size, and PVTT consistently contributed substantially to the prediction of 6-, 12-, and 24-month OS (**Figure 5**).

## Discussion

In this multicenter retrospective study, we systematically evaluated the survival benefit of adding radiotherapy to immunotherapy combined with targeted therapy in patients with BCLC stage C hepatocellular carcinoma, and further explored the role of artificial intelligence in prognostic prediction. Our results demonstrated that the addition of radiotherapy was associated with significantly prolonged progression-free survival and overall survival compared with immunotherapy plus targeted therapy alone. Importantly, this survival advantage remained consistent both before and after adjustment using inverse probability of treatment weighting, radiotherapy was associated with improved survival in this cohort when incorporated into multimodal treatment strategies for advanced HCC.

The observed survival improvement with immunoradiotherapy is supported by strong biological plausibility. Radiotherapy is increasingly recognized not only as a local cytotoxic treatment, but also as a potent modulator of antitumor immunity. Previous studies have shown that radiation can induce immunogenic tumor cell death, increase tumor antigen release, promote dendritic cell activation, and enhance antigen presentation (25, 26). In addition, radiotherapy can remodel the tumor microenvironment by upregulating immune-related molecules and reversing local immunosuppression, thereby enhancing the efficacy of immune checkpoint blockade (27, 28). In advanced HCC, which is typically characterized by a high tumor burden, vascular invasion, and an immunosuppressive microenvironment, these synergistic effects may be particularly relevant (29, 30). Nevertheless, clinical responses to immunoradiotherapy remain heterogeneous, highlighting the need for reliable methods to stratify patients and predict individual outcomes.

To address this challenge, we developed and validated multiple AI–based prognostic models integrating key clinical and tumor-related variables. These variables included radiotherapy status, tumor number, tumor size, liver function assessed by Child–Pugh class, presence of portal vein tumor thrombosis, and serum alpha-fetoprotein level. Among the evaluated machine learning algorithms, the random survival forest model demonstrated the most robust and stable predictive performance. This was evidenced by consistently favorable time-dependent area under the receiver operating characteristic curves, stable decision curve analysis indicating potential clinical usefulness, and excellent calibration, with close agreement between predicted and observed survival probabilities across multiple time points (31, 32).

Notably, variable importance analysis consistently identified tumor number, tumor size, and portal vein tumor thrombosis as the most influential predictors of overall survival at 6, 12, and 24 months. These findings emphasize that indicators of tumor burden and vascular invasion remain dominant determinants of prognosis even in the era of immunoradiotherapy (33). Although advances in systemic therapy have improved outcomes for selected patients, aggressive tumor biology continues to play a central role in survival. The consistent importance of these variables across different prediction horizons also supports their inclusion in prognostic modeling and clinical decision-making. Furthermore, correlation analysis demonstrated low inter-variable dependence, suggesting that each factor contributes independently to survival prediction and enhances model stability.

Our study highlights the value of artificial intelligence as a complement to traditional survival analysis. Conventional statistical models, such as proportional hazards regression, rely on linear assumptions and may be limited in their ability to capture complex interactions among prognostic factors. In contrast, machine learning approaches are capable of modeling nonlinear relationships and high-order interactions, allowing for more flexible and individualized risk prediction (34, 35). In the context of advanced HCC, AI–driven prognostic tools may assist clinicians in identifying patients who are most likely to benefit from aggressive combination treatments such as immunoradiotherapy, while avoiding overtreatment in patients with limited expected benefit (36).

From a clinical perspective, the RSF model may be used as a decision-support tool for risk stratification. Patients with higher RSF-predicted risk can be considered a clinically ‘high-risk’ subgroup. Identifying such patients may help clinicians implement closer surveillance and earlier response assessment, prompt multidisciplinary evaluation, and consider earlier integration or optimization of local therapy when feasible, as well as clinical trial referral where available. In addition, high-risk identification may encourage proactive optimization of liver function and supportive care to maintain treatment tolerance.

Several limitations of this study should be acknowledged. First, Although IPTW was used to balance measured baseline covariates, this retrospective observational study remains subject to residual confounding and selection bias; therefore, the observed associations should not be interpreted as causal effects. Second, the sample size was relatively modest, and all patients were derived from Chinese institutions, which may limit the generalizability of our findings to other populations. Third, the prognostic models were based primarily on clinical variables; incorporation of radiomics features, molecular biomarkers, and immune-related parameters may further enhance predictive accuracy and biological interpretability. Finally, the model was not externally validated. Independent cohorts and prospective studies are needed to confirm robustness and clinical utility before routine implementation.

In conclusion, radiotherapy use was associated with improved survival in BCLC stage C HCC treated with immunotherapy plus targeted therapy. Artificial intelligence–based prognostic modeling, particularly using random survival forest algorithms, provided stable and reliable individualized survival prediction. The integration of artificial intelligence with immunoradiotherapy represents a promising approach to advance precision medicine and optimize treatment strategies for patients with advanced hepatocellular carcinoma.

## Data Availability

The original contributions presented in the study are included in the article/**Supplementary Material**. Further inquiries can be directed to the corresponding author.
