# Supplementary material for: Artificial intelligence–based prognostic modeling of immunoradiotherapy in Barcelona clinic liver cancer stage C hepatocellular carcinoma: a multicenter retrospective study
Source: Front Oncol. 2026 Mar 5;16:1784711. doi: 10.3389/fonc.2026.1784711 (PMC12999377; doi:10.3389/fonc.2026.1784711)

Supplementary Fig. 1. Standardized mean difference (SMD) of baseline covariates before and after inverse probability of treatment weighting (IPTW).


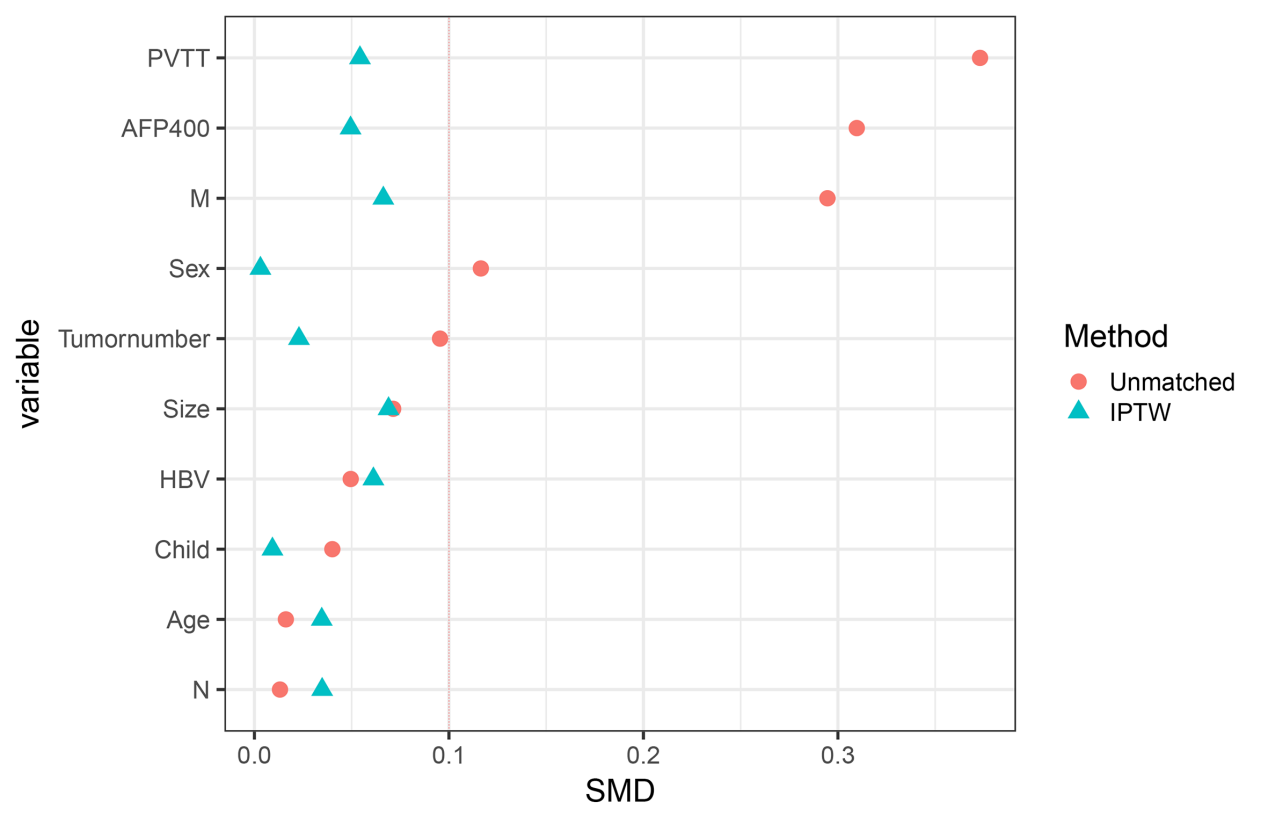

Supplement: Supplementary file 1 [file DataSheet1.docx]
